# Ultrasound and Microbubbles Enhance Uptake of Doxorubicin in Murine Kidneys

**DOI:** 10.3390/pharmaceutics13122038

**Published:** 2021-11-29

**Authors:** Oystein Eikrem, Spiros Kotopoulis, Mihaela Popa, Mireia Mayoral Safont, Kjell Ove Fossan, Sabine Leh, Lea Landolt, Janka Babickova, Oddrun Anita Gudbrandsen, Odd Helge Gilja, Bettina Riedel, Jan Schjøtt, Emmet McCormack, Hans-Peter Marti

**Affiliations:** 1 Department of Clinical Medicine, University of Bergen, 5021 Bergen, Norway; oystein.eikrem@uib.no (O.E.); sabine.leh@helse-bergen.no (S.L.); lea.landolt@uib.no (L.L.); jana.babickova@gmail.com (J.B.); oddrun.gudbrandsen@uib.no (O.A.G.); odd.gilja@uib.no (O.H.G.); hans-peter.marti@uib.no (H.-P.M.); 2 Department of Medicine, Haukeland University Hospital, 5021 Bergen, Norway; 3 National Centre for Ultrasound in Gastroenterology, Haukeland University Hospital, 5021 Bergen, Norway; 4 Neoety AS, 2040 Kløfta, Norway; 5 Department of Clinical Science, University of Bergen, 5021 Bergen, Norway; Mihaela.popa@uib.no (M.P.); mireia.safont@uib.no (M.M.S.); bettina.riedel@helse-bergen.no (B.R.); jan.didrik.schjott@helse-bergen.no (J.S.); emmet.mc.cormack@uib.no (E.M.); 6 Department of Medical Biochemistry and Pharmacology, Haukeland University Hospital, 5021 Bergen, Norway; kjell.ove.fossan@helse-bergen.no; 7 Department of Pathology, Haukeland University Hospital, 5021 Bergen, Norway; 8 Institute of Molecular Biomedicine, Comenius University, 81108 Bratislava, Slovakia

**Keywords:** ultrasound, microbubbles, sonoporation, doxorubicin, kidney, mice

## Abstract

The use of ultrasound and microbubble-enhanced drug delivery, commonly referred to as sonoporation, has reached numerous clinical trials and has shown favourable results. Nevertheless, the microbubbles and acoustic path also pass through healthy tissues. To date, the majority of studies have focused on the impact to diseased tissues and rarely evaluated the impact on healthy and collateral tissue. The aim of this study was to test the effect and feasibility of low-intensity sonoporation on healthy kidneys in a mouse model. In our work here, we used a clinical diagnostic ultrasound system (GE Vivid E9) with a C1-5 ultrasound transducer combined with a software modification for 20-µs-long pulses to induce the ultrasound-guided drug delivery of doxorubicin (DOX) in mice kidneys in combination with SonoVue^®^ and Sonazoid™ microbubbles. The acoustic output settings were within the commonly used diagnostic ranges. Sonoporation with SonoVue^®^ resulted in a significant decrease in weight vs. DOX alone (*p* = 0.0004) in the first nine days, whilst all other comparisons were not significant. Ultrasound alone resulted in a 381% increase in DOX uptake vs. DOX alone (*p* = 0.0004), whilst SonoVue^®^ (*p* = 0.0001) and Sonazoid™ (*p* < 0.0001) further increased the uptake nine days after treatment (419% and 493%, respectively). No long-standing damage was observed in the kidneys via histology. In future sonoporation and drug uptake studies, we therefore suggest including an “ultrasound alone” group to verify the actual contribution of the individual components of the procedure on the drug uptake and to perform collateral damage studies to ensure there is no negative impact of low-intensity sonoporation on healthy tissues.

## 1. Introduction

The combination of ultrasound and microbubbles to improve the local drug uptake at a given location is commonly known as sonoporation [1]. Several publications have shown therapeutic efficacy utilizing sonoporation both in vitro and in vivo [2,3,4,5,6]. Examples of high-intensity (>100 W/cm^2^) [7] and low-intensity (<1 W/cm^2^, typically within the diagnostic range) [8] ultrasound settings have been shown as effective in enhancing drug uptake. Depending on the intensity of the ultrasound, the mechanism of action of sonoporation can vary and be complex. In general, sonoporation is induced when ultrasound waves force microbubbles at a target site to rapidly expand and contract millions of times per second. These “oscillating” microbubbles interact with the nearby cells, primarily vascular endothelial cells, forming microscopic pores [9] or forcing tight intercellular junctions to open [10,11]. This allows more drugs to be delivered to the target tissue. We have previously shown preclinical benefits of sonoporation at low acoustic intensities [12]. A phase I human clinical trial has also been conducted evaluating the treatment of inoperable adenocarcinoma of the pancreas where ultrasound and microbubbles combined with gemcitabine significantly extended their survival [13]. Recently, the use of ultrasound with and without microbubbles to target the kidneys was highlighted as a potential promising novel treatment modality [14].

Whilst ultrasound can be accurately targeted in vitro with submillimetre precision, even when targeting deep tissues, the ultrasound still needs to propagate through the tissue between the ultrasound transducer and target, i.e., potentially other healthy organs and tissues. Furthermore, human tissues, unlike water, are very heterogenous, meaning the ultrasound can be diffracted and scattered into other nearby tissue. A common way to compensate for these issues is to use a large aperture transducer with a high focusing power, but this still means healthy tissue along the acoustic path may be subjected to lower-intensity ultrasound. The impact on such healthy tissues is rarely evaluated and may have a large impact on how sonoporation may be performed in a clinical setting.

The aim of this study was to test the feasibility and impact of sonoporation on healthy tissue (kidneys) as a model for collateral tissue by evaluating the serum creatinine, kidney histology, and uptake of doxorubicin and doxorubicinol. Although doxorubicin is mostly used to treat cancer, it has been extensively investigated as a model drug in the field of nephrology based on its nephrotoxicity causing focal and segmental glomerulosclerosis [15]. To our knowledge, this is the first study that has evaluated the impact of sonoporation on healthy mice kidneys whilst comparing two microbubble formulations and quantifying the concentration of drug delivered to the tissue over multiple time points.

## 2. Materials and Methods

**Figure 1:**
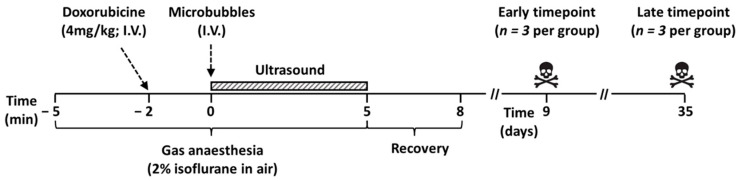
Timeline of the experimental setup. Mice were anesthetised five minutes prior to the ultrasound treatment. Doxorubicin was injected two minutes prior to the ultrasound treatment, and the microbubbles were injected immediately before ultrasound application, which lasted for five minutes. Mice were kept in a UV-heated chamber for recovery for approximately three minutes. Mice were sacrificed after 9 or 35 days.

A software-modified clinical diagnostic ultrasound system was used to induce image-guided sonoporation in both kidneys in immunodeficient mice using two types of microbubbles. Acute toxicity was evaluated by comparing the body weights. The tissue concentrations (consisting of intracellular, extracellular, and microcirculatory levels) of doxorubicin and its active metabolite doxorubicinol were measured using high-performance liquid chromatography–tandem mass spectrometry (HPLC-MS/MS) [16]. Histological images were used to evaluate the long-term cellular damage at the end of the study. The timeline of the experimental procedure can be delineated from Figure 1.

### 2.1. Animals

All experiments were approved by The Norwegian Food Safety Authority (Approval No.: 16/159013, Valid from: 02 January 2017) and conducted according to The European Convention for the Protection of Vertebrates Used for Scientific Purposes. NOD-scid IL2rγ^null^ male mice (Gades Institute, University of Bergen; originally a generous gift of Prof. Leonard D. Shultz, Jackson Laboratories, Bar Harbour, ME, USA) were used for this study. Animals were randomly allocated to experimental groups and kept in separate cages to avoid ingestion of faecal material that may contain drugs. A total of 24 mice were randomly divided into four groups of 6 (Table 1). The minimum number of animals was used for this study to comply with the 3Rs of ethical research and EU directive (Directive 2010/63/EU, 2010). This mouse strain used as our group has previously shown that it is susceptible to sonoporation in a pancreatic cancer model [17] and for a breast cancer model by others [18].

### 2.2. Doxorubicin and Maximum Tolerated Dose

Doxorubicin has been proven to be highly distributed to the kidneys [19] and serves as a model drug for evaluating drug uptake to the kidneys in some murine models and was therefore selected as the model drug for this investigation [20].

An a priori maximum tolerated dose (MTD) study was performed to determine the optimal doxorubicin dose. Body weight and animal appearance were observed daily to determine toxicity. Single-dose injections of 5, 7.5, 10, and 12.5 mg/kg were evaluated [20]. All doses resulted in weight loss as a sign of acute toxicity. The lowest dose of 5 mg/kg resulted in an acceptable weight loss followed by weight gain. As the addition of sonoporation treatment is expected to increase animal stress, the dose was reduced to 4 mg/kg. This dose was sufficient to allow the detection of both doxorubicin and its metabolite in tissue samples.

### 2.3. Ultrasound and Microbubble Treatment

**Figure 2:**
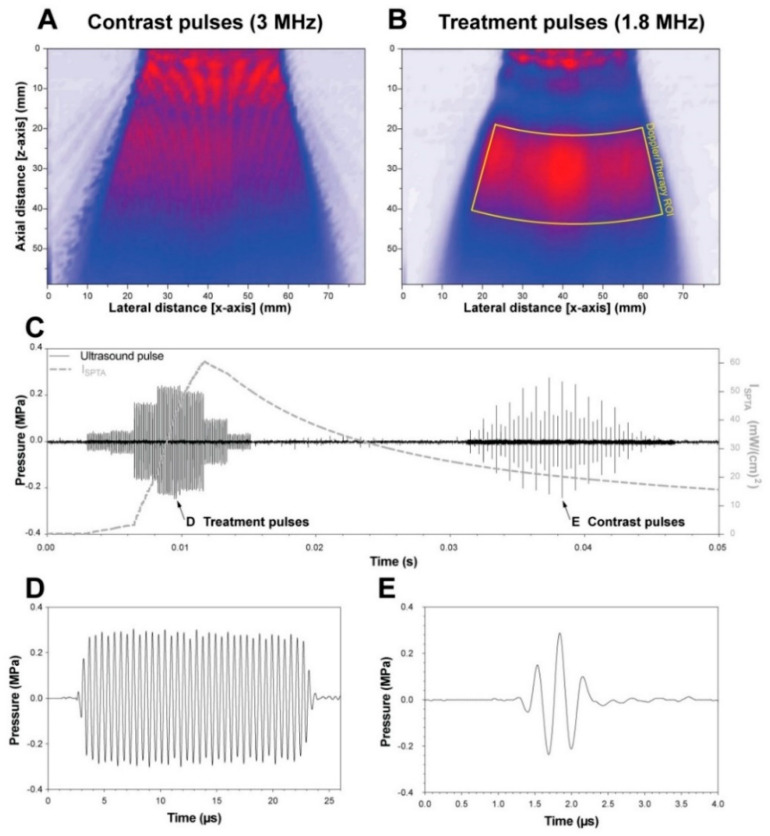
Ultrasound field calibrations using a needle hydrophone. (**A**,**B**) A representative normalised 2D pressure distribution of the contrast imaging pulses (**A**) and 20-µs treatment pulses (**B**) over an entire frame. The red–blue–white colour gradient indicates pressure, where red is the maximum pressure, blue is the half-maximum, and white is no signal. (**C**) The pressure waveform over an entire frame at the expected treatment depth, where the left pulse train is 12 packets of 20-µs pulses, and the right pulse train is the phase inversion contrast imaging. (**D**) There is a single 20-µs treatment pulse and (**E**) a single contrast imaging pulse.

Ultrasound was generated by a C1-5 transducer connected to an E9 clinical diagnostic ultrasound system with a research software package (supplied by GE Global Research, Niskayuna, NY, USA). The research package extends the ability to control the transmit frequency and pulse lengths and to interleave them with diagnostic imaging pulses. This is achieved via duplex imaging enabling a contrast imaging/colour doppler imaging configuration where the colour doppler pulses are replaced with long “treatment” pulses. The contrast image used a centre frequency of 3.0 MHz and 3 cycles in phase inversion/amplitude modulation configuration. The treatment pulse was set at 20 µs and had a centre frequency of 1.8 MHz. The beam density was set to high, and the packet size was set to 12. The ultrasound field was calibrated in a custom-made, motorised, 3-axis water tank using a 200-µm needle hydrophone kit (NH0200, Precision Acoustics Ltd., Dorchester, UK) using a traditional snake raster with a 500-µm step size in both axes. Following calibration in a water tank, the ultrasound transmission settings were set to achieve a peak-negative pressure of 0.25 MPa without attenuation. The attenuation of the mouse skin at 2.25 MHz was 5.19 dB/cm [21]. The mouse skin was approximately 0.200 cm thick, resulting in an attenuation of 1.04 dB (11.3% ultrasound loss) [22]. Hence, an attenuation of 11.3% was used when calculating the acoustic output. This resulted in an in situ pressure of 0.222 MPa, i.e., a Mechanical Index (MI) of 0.165 for the 1.8 MHz treatment pulse and MI of 0.128 for the 3.0-MHz contrast mode pulse, resulting in an on-screen Thermal Index for soft tissues (TIs) of 0.2 when combined. The spatial peak, temporal average intensity of the entire pulse train was 16 mW/cm^2^. The sector width of the image (i.e., active portion of the ultrasound transducer) was adjusted to ensure only the portion in contact with the mouse was active to minimise the acoustic field distortion from the areas of no tissue. The largest transmit beam aperture was less than 1.0 cm; hence, even if only a part of the transducer was in contact with the animal, an effective acoustic deposition was expected. An interpolated, attenuation-free, 2D hydrophone scan representation of the acoustic fields generated for the contrast and treatment pulses can be seen in Figure 2A,B, respectively. These images were reconstructed in MATLAB 2018a (MathWorks, Natick, MA, USA) after deconvolving the received hydrophone signal with the calibrated hydrophone frequency response for each scan point. The acoustic components of the contrast and treatment pulses (Figure 2A,B) were separated by using a bandpass filter at 2.4 MHz. An example of the pressure waveform and interleave pattern can be seen in Figure 2C–E. The frame rate was maximised by reducing the imaging depth and the ROI reaching a value of 17 frames per second. The multiple pulses of various amplitudes (seen in Figure 2C) in the treatment and contrast pulse trains were due to the multiple acoustic beams generated by the beam scan pattern in a clinical diagnostic scanner, i.e., the low-amplitude pulses to the left and right of the pulse of maximum amplitude were from the ultrasound beams to the left and right (c.f., red lines in Figure 2A) of the ultrasound beam currently being measured.

We tested two different types of commercially available microbubbles: Sonazoid^TM^ (GE Healthcare, Little Chalfont, UK) and SonoVue^®^ (Bracco Imaging S.p.A., Milano, Italy). Sonazoid^TM^ was diluted 4-fold to match the concentration of SonoVue^®^ as measured in-house using optical microscopy. Microbubbles were used within 20 min of reconstitution. Table 2 presents an overview of the physicochemical characteristics of SonoVue^®^ and Sonazoid™.

Five minutes prior to treatment, mice were anesthetised using 5% isoflurane (Isoba^®^ vet, Intervet, Kenilworth, NJ, USA) in air to prevent modifying the oxygen window and reducing the microbubble stability. Mice were kept anesthetised until the end of the treatment using 2% isoflurane in air. Abdominal hair was first shaved, and the remaining fine hairs were removed using depilatory cream (Veet^®^, Reckitt Benckiser Group, Slough, UK). Mice were injected with 4 mg/kg of doxorubicin <2 min prior to ultrasound treatment to best match the peak blood–plasma concentration during ultrasound application based on previous pharmacokinetics studies that have demonstrated the highest plasma concentration at the earliest sampling time point [27]. A 50-µL bolus of microbubbles (or saline in the control group) was injected into the tail vein using a 29-G needle (B. Braun, Melsungen, Germany) immediately prior to the ultrasound treatment. A bolus injection of microbubbles was used to mimic an effective sonoporation configuration, as seen in previous preclinical studies [17,28] and clinical studies [13].

**Figure 3:**
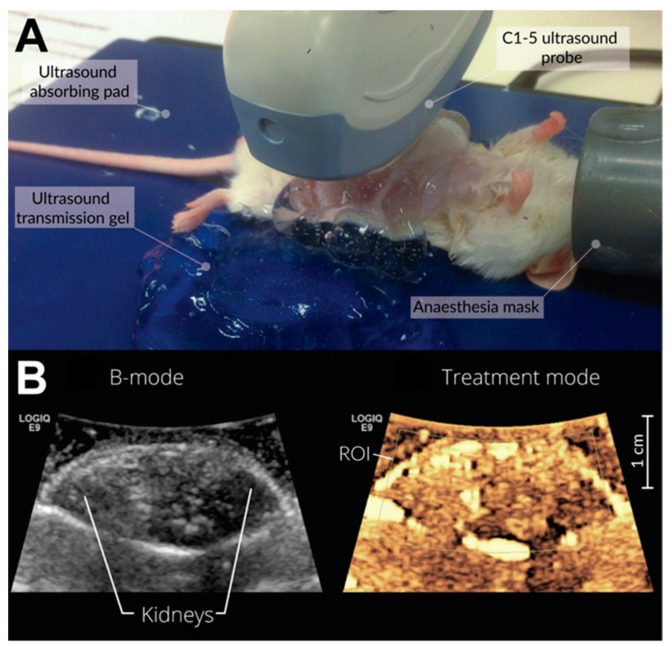
Photograph of treatment configuration and a representative ultrasound image. Mice were placed in a dorsal recumbent position on a heated, ultrasound transmission gel-covered ultrasound absorption pad, and the C1-5 curvilinear probe was placed on the abdomen to target both kidneys (**A**). Exact alignment of the kidneys was achieved via the ultrasound image where the kidneys could be clearly delineated (**B**, **left**). The treatment mode (**B**, **right**) is a contrast mode image, captured prior to microbubble injection, that shows the locations of nonlinear echoes. The bright areas seen in this image are due to high-amplitude echoes that also contain nonlinear acoustic contents. The ROI indicates the focal area where long treatment pulses are targeted and where treatment is expected.

During ultrasound treatment, the mice were placed in the dorsal recumbent position on a heated, ultrasound transmission gel-covered ultrasound absorption pad. The surface temperature was set to 37 °C. The C1-5 curvilinear probe (GE Global Research, Niskayuna, NY, USA) was placed in a perpendicular position imaging both kidneys (Figure 3A). The ultrasound probe, the treatment region, was aligned using a B-mode ultrasound image (Figure 3B, left panel). During treatment, the system was switched to contrast imaging with Doppler. Hence, only the contrast image could be observed during treatment (Figure 3B, right panel). The ROI determined the focal position and the area where the 20-µs pulses (therapeutic pulses) were targeted toward (Figure 3B, right panel), i.e., the area where the ultrasound-enhanced therapy was expected. The mice were treated with ultrasound for a total of 5 min. After treatment, the anaesthesia was immediately turned off, and mice were placed in a UV-heated chamber for 3–5 min until awake and recovered.

### 2.4. Toxicity Evaluation

Body weight measurements were the primary method used to evaluate the toxicity following treatment [29,30]. A decrease in body weight indicated increased toxicity, correlating to increased drug uptake. A body weight loss surpassing 20% of the pre-treatment body weight was set as the threshold for toxicity [31].

### 2.5. Drug Delivery and Uptake: Doxorubicin and Doxorubicinol Measurements

Doxorubicin and its cardiotoxic metabolite doxorubicinol were quantified using HPLC-MS/MS (Agilent Technologies 1290 Infinity LC System and Agilent Technologies 6490 Triple Quadrupole LC-MS System, Santa Clara, CA, USA). Following euthanasia on day 9 and day 30, the kidneys were extracted and rinsed carefully in clean phosphate-buffered saline and snap-frozen in liquid nitrogen. Samples were collected at days 9 and 30 to evaluate the long-term impact of sonoporation and the time required for the tissue to return to baseline. The kidneys were stored at −80 °C until further analyses. The entire kidneys were analysed except for the middle cross-section that was used for histology. The whole kidneys were weighed and placed in a 2-mL centrifuge tube with a screw cap. A total of 300 µL of Milli-Q water with a concentration of 73 nMol/L of internal standard daunorubicin was added. Porcelain beads were used to homogenise the tissue using a Precellys 24 apparatus (Bertin Instruments, Bretonneux, France) set at the speed of 6800 rpm. The homogenisation process was repeated three times for 10 s each. Following homogenisation, 100 µL of 1-M Trizma buffer (Sigma-Aldrich, St. Louis, MO, USA) (pH 11.1) was added to the samples. After mixing, 1 mL of ethylacetate/heptane was added and vortexed for one minute. The samples were centrifuged for six min at 10,000× *g*. The organic phase was transferred to a new tube, and the samples were evaporated until dried at 50 °C in N_2_ gas. To dissolve the samples, 25 µL of methanol and 25 µL of water were added. The samples were transferred to a 96-well microtiter plate (Greiner Bio-One GmbH, Kremsmünster, Austria) for analysis. An injection volume of 20 µL and flow set to 400 µL per minute were used. The mobile phases were acetonitrile and 0.1% formic acid in water. Kinetex Biphenyl 2.1 × 50-mm columns with 2.7-µm particles were used (Phenomenex, Torrance, CA, USA). A detection range limit for doxorubicin and doxorubicinol of around 0.4 pMol/g of tissue was achieved.

### 2.6. Histology

Almost the entire kidney was used for the tissue drug analyses; however, the middle transverse section of the kidneys was cut out and fixed with formalin and embedded in paraffin. Tissue sections with a thickness of 2 to 3 µm were stained using the Periodic acid-Schiff (PAS) staining method. Slides were scanned with Aperio ScanScope^®^ XT (Leica Biosystems, Wetzlar, Germany) at 40×/1.0NA objective magnification and viewed in ImageScope 12 (Leica Biosystems, Wetzlar, Germany). The images were analysed for signs of nephrotoxicity, i.e., tubular injury and evidence of doxorubicin nephropathy, by a blinded and experienced consultant nephropathologist.

### 2.7. Kidney Function

Kidney function was evaluated by measuring the serum creatinine with a Cobas c 111 apparatus (Roche Diagnostics, GmbH, Basel, Switzerland). Whilst a reference cut-off value for the presence of kidney damage has not been well-established, using this method for groupwise comparisons still stands. Higher concentrations of serum creatinine indicate a higher degree of kidney damage. For these serum analyses, we pooled the samples from 9 days and 35 days due to the small sample size.

### 2.8. Statistical Analyses

Statistical comparisons between groups were performed with one-way ANOVA with multiplicity adjusted *p*-values (Dunnett’s multiple comparisons test). The statistical comparisons of the weight changes were done with the repeated measures two-way ANOVA method and with Dunnett’s post hoc test. A *p*-value of <0.05 was considered significant. Statistical analyses were performed using Prism 6.05 for Windows (GraphPad Software, Inc., La Jolla, CA, USA).

## 3. Results and Discussion

### 3.1. Body Weight

**Figure 4:**
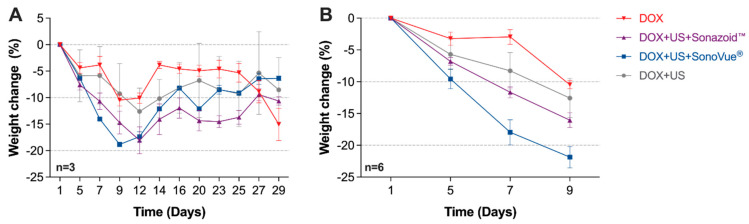
Percentage of weight change after a single treatment on day 1. During the first 2 weeks, there were signs of increased toxicity with increased weight loss in the groups receiving DOX + US + microbubbles (**A**) (*n* = 3) for the whole study period and (**B**) (*n* = 6) until day 9. DOX = Doxorubicin; US = Ultrasound.

Five days after a single DOX treatment, all four groups showed weight loss. This trend continued until days 12–14; after which, all groups started to recover (Figure 4A). The DOX + US + Sonazoid^TM^ and the DOX + US + SonoVue^®^ groups lost the most weight initially. In a two-way repeated measures ANOVA, there were overall significant differences in the weight change (*p* < 0.0001). In the post hoc Bonferroni’s test comparing the whole study period, there were no statistically significant differences comparing all four groups against each other for the three non-sacrificed mice (Figure 3A). During the first nine days (six mice per group), there were statistically significant weight differences, as established from both the overall test and the post hoc test comparing DOX alone vs. DOX + US + SonoVue^®^ (*p* = 0.0004) and not significant comparing DOX alone and DOX + US + Sonazoid^®^ (*p* = 0.056). The higher weight loss during the first two weeks in the DOX + US + Sonazoid^®^ and the DOX + US + SonoVue^®^ groups could be explained by a higher acute toxicity [32]. As all the groups were anaesthetised for the same period, the weight loss difference between DOX alone and treated groups is not expected to be due to increased stress induced by the anaesthesia procedure but, rather, the impact of the sonoporation with DOX.

Based on the summary of the product characteristics given by the European Medicines Agency, doxorubicin has a final half-life of 30 h. Thus, it would be theoretically eliminated within 10 days, with some contribution due to the metabolite. Therefore, the main effect on weight (and differences between groups) would be expected to take place during the first two weeks after injection.

### 3.2. Doxorubicin Uptake and Its Metabolite

**Figure 5:**
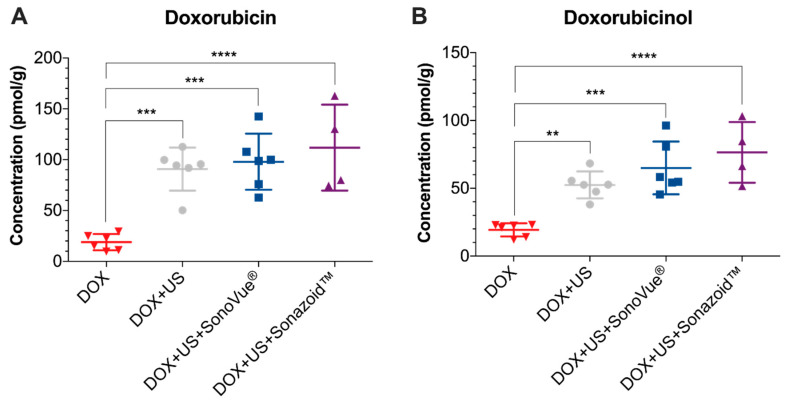
Concentrations (euthanasia day 9) of doxorubicin (**A**) and doxorubicinol (**B**). One-way ANOVA Dunnett’s multiple comparisons test with multiplicity adjusted *p*-values comparing the control group with all the treatment groups. ** *p*-value < 0.01, *** *p*-value < 0.001, and **** *p*-value < 0.0001.

The measurements of doxorubicin/doxorubicinol concentrations in homogenised kidney tissue lysates (euthanasia day 9) showed substantially higher concentrations in the ultrasound treatment groups when compared to the control (DOX) group (Figure 5). Ultrasound alone significantly increased the uptake of doxorubicin from a mean of 18.9 pmol/g to 90.8 pmol/g (*p* = 0.0004). Several studies supported the increased uptake with just the application of ultrasound alone but not at such low acoustic pressures [33,34]. The addition of microbubbles further increased the uptake of doxorubicin up to 97.9 pmol/g for SonoVue^®^ (*p* = 0.0001 vs. DOX) and 111.8 pmol/g for Sonazoid^TM^ (*p* < 0.0001 vs. DOX) (Figure 5A). There was no significant difference between DOX + US and either of the microbubble groups. Similar results were seen for doxorubicinol (Figure 5B). Ultrasound alone significantly increased the uptake of doxorubicinol into the kidney tissue from a mean of 19.3 pmol/g to 52.5 pmol/g (*p* = 0.0032). The addition of microbubbles further increased the uptake of doxorubicinol to 65.0 pmol/g for SonoVue^®^ (*p* = 0.0001 vs. DOX) and 76.4 pmol/g for Sonazoid^TM^ (*p* < 0.0001 vs. DOX). There was no significant difference between DOX + US and the microbubble groups for the metabolite. After five weeks, the doxorubicin and doxorubicinol concentrations were below the detection limit.

The present results of the enhanced uptake of doxorubicin and doxorubicinol in kidney tissue are promising. The data also suggest that doxorubicin and doxorubicinol did not “escape”, e.g., through the transient pores that are assumed to be formed during sonoporation [35]. Hence, the increased drug delivery should likely have an increased therapeutic effect at the target organ.

However, our results contrast with other publications that indicated that the addition of microbubbles and ultrasound, when compared to ultrasound alone, should further increase the drug uptake. A reason might be that the tissue samples were collected as late as 9 days after a single treatment. Therefore, a higher uptake at an earlier stage with the addition of microbubbles might not have been detected. The ultrasound conditions used here may not have been optimal for the microbubbles due to low acoustic pressure or suboptimal ultrasound frequency for an in vivo dynamic model. The drug uptake due to ultrasound alone may be due to shear stress induced by the long ultrasound pulses. Mazzawi et al. showed that, even at low peak-negative pressures (0.2 MPa), ultrasound without microbubbles can alter cell signalling pathways, inhibiting the motility and modulating the morphology of Madin-Darby Canine Kidney cells in vitro [36]. Furthermore, numerous studies [37] using ultrasound alone have been shown to induce cellular changes that could result in increased drug uptake, which is hypothesised to be due to cells themselves being acoustically responsive in a similar manner as microbubbles, the increased generation of reactive oxygen species, free radicals, or increased calcium uptake [38].

### 3.3. Kidney Histology

**Figure 6:**
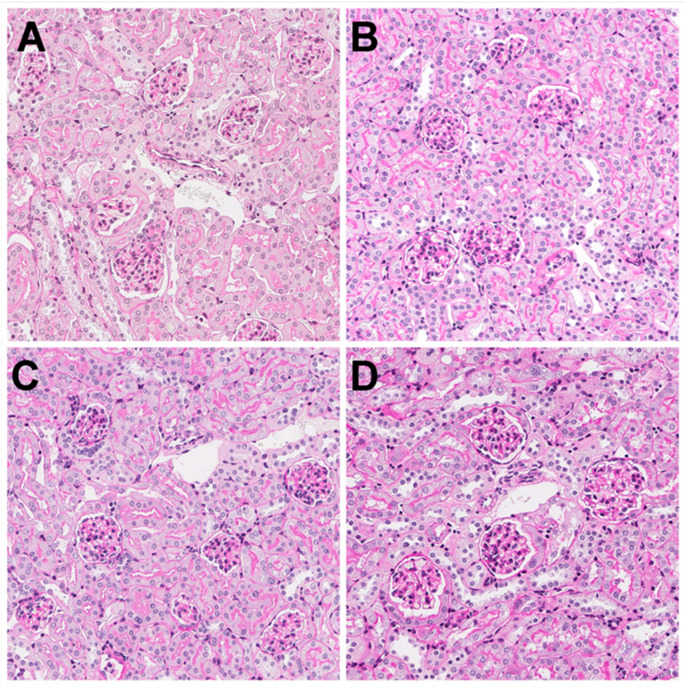
Light microscopy image of a kidney histology sample using Periodic acid-Schiff staining. (**A**) doxorubicin; (**B**) doxorubicin and ultrasound; (**C**) doxorubicin, ultrasound, and Sonazoid™; and (**D**) doxorubicin, ultrasound, and SonoVue^®^. All four groups had normal-looking renal structures. Picture size: 400 µm × 400 µm at 20× magnification.

The histological analysis displayed no difference between the groups in samples taken from the kidney tissues at day 35 (late time point). A representative example of a histological section from each of the four groups is shown in Figure 6. All the images display normal-looking glomerular and tubular structures without any visible tissue damage. These results indicate that, whilst there may have been an increased drug uptake, the current ultrasound settings did not induce tissue damage that resulted in permanent tissue scarring. If tissue damage occurred during the treatment, the mice showed complete recovery, indicating the safety of this technique. However, the animals did show clear signs of malaise and substantial weight loss, both in the initial dose-finding study and in this pilot study (Figure 4). In this pilot study, we did not address cardiotoxicity or bone marrow depletion, amongst other adverse effects.

### 3.4. Kidney Function

**Figure 7:**
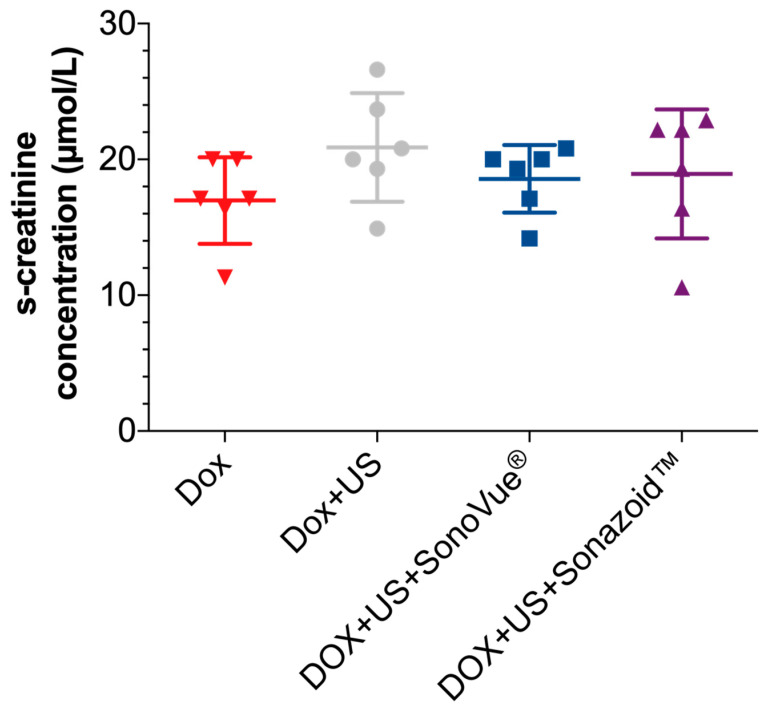
Serum creatinine measurements at day 9 and day 35 combined after treatment. Although higher creatinine concentrations were detected in the treatment groups, they were not significantly higher than in the control group.

Serum creatinine is a marker with the ability to detect kidney damage [39]. Serum creatinine was measured for all the animals. A mean concentration (±SD) of 17.0 ± 3.2, 20.9 ± 4.0, 18.6 ± 2.5, and 18.9 ± 4.8 µMol/L was measured for the DOX, DOX + US, DOX + US + SonoVue^®^, and DOX + US + Sonazoid^TM^ groups, respectively (Figure 7). One-way ANOVA with Dunnett’s multiple comparisons test with multiplicity adjusted *p*-values did not detect any significant differences between the four groups. However, sonoporation did not alter the renal tissue structure and function as measured by histology or serum creatinine, respectively. Yet, a respective effect on the more sensitive analysis of kidney function—namely, the measured glomerular filtration rate—cannot be excluded. Interestingly, our results suggest that sonoporation could enhance the uptake of a cytotoxic drug with a known high distribution to kidney tissue in the first place.

### 3.5. Study Limitations and Future Work

In this study we also used low-intensity sonoporation with an MI < 0.2. Inertial, i.e., the violent collapse of microbubbles, is not expected at these acoustic conditions; rather, stable cavitation where the microbubbles rapidly oscillate without collapsing is expected. SonoVue^®^ and Sonazoid™ are expected to undergo inertial cavitation at peak-negative pressures above 0.4 MPa [40,41], whereas the acoustic pressures used in this study were approximately half that. The use of stable cavitation to induce sonoporation has been shown before [17,42,43]. At these low-acoustic energy levels, an ultrasound-induced temperature rise of 0.2 °C (i.e., the TIs) is expected as the worst-case scenario; hence, ultrasound-induced hyperthermia is not expected to be the root cause of the drug delivery. Other studies have shown that the lipid transfer between the microbubble shell and cell membrane may enhance the cell permeability and may be the cause behind the observed uptake [44], but in our work here, increased drug delivery was also seen with ultrasound alone. Hence, such a mechanism would only be part of the difference between the US + DOX and US + DOX + microbubble groups. In this study, we used a bolus injection of microbubbles that did not have a steady state; rather, it had a rapid peak followed by a rapid decrease in the bubble concentration, meaning that the optimal microbubble concentration was not present for a sufficient amount of time. This variable concentration may be a reason for the small difference between the microbubble and no microbubble groups, but in our previous study, a bolus still induced a significant therapeutic effect, albeit in a less-perfused tissue (pancreatic cancer) [17].

Whilst there was no significant difference between SonoVue^®^ and Sonazoid™, the microbubbles had different physicochemical properties (Table 2). These differences will result in the two microbubble formulations having a different volumetric oscillation amplitude from the same ultrasound field, meaning that optimisation of the ultrasound pulse (i.e., tuning the frequency and bandwidth to better match the microbubble size distribution) may further improve the drug uptake.

A variable that was not addressed in this study was the order of injection of the agents, i.e., if the doxorubicin was injected after the microbubbles or after the ultrasound, a different result may have been obtained. Such an experiment may also help elucidate the mechanism of action.

Considering the serum half-life of doxorubicin in various pharmacokinetic animal studies [16,45,46], very low concentrations of the remaining drug would be expected at 9 days. Despite that, both doxorubicin and doxorubicinol were detectable at concentrations far beyond the detection limit. It is known that both doxorubicin and its metabolite can accumulate in several tissues, including the heart, liver, and kidneys [47]. Although accumulation can take place after a single injection of 12 mg/kg of doxorubicin, Johansen et al. found a four-fold decrease in the kidney tissue concentrations after just 24 h [45]. Nevertheless, our results showed significant differences in the drug and metabolite concentrations vs. the control group, indicating that there also might be a long-term effect of sonoporation.

Future studies should carefully select suitable time points for tissue concentration measurements and relate these to more sensitive kidney toxicity indices.

## 4. Conclusions

Our study showed that the application of ultrasound with or without microbubbles might enhance the delivery of doxorubicin to mouse kidney tissues without inducing longstanding damage. This increased drug uptake was detectable nine days after treatment. Higher concentrations of doxorubicin and its metabolite in kidney tissue were associated with sonoporation, and the addition of microbubbles increased the weight loss. Our study suggests that ultrasound itself, at near-diagnostic conditions, might increase the drug uptake. In future sonoporation studies with bigger sample groups, we therefore suggest including an “ultrasound alone” group to verify the actual contribution of the individual components of the procedure on the drug uptake. Furthermore, tissue drug concentrations immediately after the application of ultrasound would be of interest in kidney tissues.

## Figures and Tables

**Table 1:** Experimental groups and posttreatment evaluation techniques and time points. DOX = Doxorubicin; US = Ultrasound.

	Posttreatment Analysis	
Treatment Regimen	Day 9 (Early Time Point) *n* = 3	Day 35 (Late Time Point) *n* = 3
DOX (*n* = 6)	Histology Drug and metabolite quantification Serum creatinine	Histology Serum creatinine
US + DOX (*n* = 6)		
US + DOX + Sonazoid^TM^ (*n* = 6)		
US + DOX + SonoVue^®^ (*n* = 6)		

**Table 2:** Physicochemical characteristics of the two ultrasound contrast agents used in this study with study references in the brackets.

Microbubble	Manufacturer	Stock Concentration (×10^8^ ppmL)	Mean Diameter (µm)	Resonance Frequency (MHz)	Shell Elasticity (Nm^−1^)
SonoVue^®^	Bracco Imaging S.p.A	2.5 [23]	2.5 [23]	3.0 [23]	0.22 [24]
Sonazoid™	GE Healthcare	12.0 [25]	2.1 [25]	4.3 [25]	0.53 [26]
